# Supplementary material for: Mechanisms governing the pioneering and redistribution capabilities of the non-classical pioneer PU.1
Source: Nat Commun. 2020 Jan 21;11:402. doi: 10.1038/s41467-019-13960-2 (PMC6972792; doi:10.1038/s41467-019-13960-2)
Supplement: Supplementary file 7 — Source data [file 41467_2019_13960_MOESM7_ESM.zip › Source_Data/FigureS2/FigureS2B_MotifScanOutput/KmeansCluster2/homerResults/motif3.similar.html]

motif3

## Information for motif3

A
C
G
T
A
G
T
C
A
G
T
C
A
G
T
C
A
G
T
C
A
G
C
T
A
C
G
T
  
Reverse Opposite:  

C
G
T
A
C
T
G
A
T
C
A
G
C
T
A
G
A
C
T
G
C
T
A
G
C
G
T
A
  

|  |  |
| --- | --- |
| p-value: | 1e-442 |
| log p-value: | -1.018e+03 |
| Information Content per bp: | 1.770 |
| Number of Target Sequences with motif | 3026.0 |
| Percentage of Target Sequences with motif | 60.24% |
| Number of Background Sequences with motif | 11263.1 |
| Percentage of Background Sequences with motif | 27.71% |
| Average Position of motif in Targets | 100.6 +/- 46.9bp |
| Average Position of motif in Background | 98.7 +/- 80.5bp |
| Strand Bias (log2 ratio + to - strand density) | 0.1 |
| Multiplicity (# of sites on avg that occur together) | 1.44 |
| Motif File: | file (matrix) reverse opposite |

### Similar de novo motifs found

|  |  |  |  |  |  |  |  |
| --- | --- | --- | --- | --- | --- | --- | --- |
| Rank | Match Score | Redundant Motif | P-value | log P-value | % of Targets | % of Background | Motif file |
| 1 | 0.891 | A G T C C G A T A G C T A G T C A G C T A G T C G A T C A C G T | 1e-178 | -410.238495 | 43.80% | 24.15% | motif file (matrix) |
| 2 | 0.772 | T A C G C T G A A C T G T C A G T C G A T C A G T A C G T C G A A C T G C T A G C T G A | 1e-167 | -385.845573 | 22.89% | 8.83% | motif file (matrix) |
